# Developmental Disruption of GABA_A_R-Meditated Inhibition in Cntnap2 KO Mice

**DOI:** 10.1523/ENEURO.0162-17.2017

**Published:** 2017-09-21

**Authors:** Morgan S. Bridi, Su Mi Park, Shiyong Huang

**Affiliations:** Program in Neuroscience, Hussman Institute for Autism, Baltimore, MD 21201

**Keywords:** autism, Cntnap2, developmental disorder, phasic inhibition, tonic inhibition

## Abstract

GABA released from presynaptic sites induces short-lived phasic inhibition mediated by synaptic GABA_A_ receptors (GABA_A_Rs) and longer-duration tonic inhibition mediated by extrasynaptic GABA_A_ or GABA_B_ receptors (GABA_B_Rs). A number of studies have found that contactin-associated protein 2 (Cntnap2) knockout (KO) mice, a well-established mouse model of autism, exhibit reduced interneuron numbers and aberrant phasic inhibition. However, little is known about whether tonic inhibition is disrupted in Cntnap2 KO mice and when the disruption of inhibition begins to occur during postnatal development. We examined tonic and phasic inhibition in layer 2/3 pyramidal cells of primary visual cortex of Cntnap2 KO at two different developmental stages, three to four and six to eight weeks of age. We found that both phasic inhibition and GABA_A_R but not GABA_B_R-mediated tonic inhibition was reduced in pyramidal cells from six- to eight-week-old Cntnap2 KO mice, while in three- to four-week-old mice, no significant effects of genotype on tonic or phasic inhibition was observed. We further found that activation of tonic currents mediated by δ-subunit-containing GABA_A_Rs reduced neural excitability, an effect that was attenuated by loss of Cntnap2. While the relative contribution of tonic versus phasic inhibition to autism-related symptoms remains unclear, our data suggest that reduced tonic inhibition may play an important role, and δ-subunit-containing GABA_A_Rs may be a useful target for therapeutic intervention in autism.

## Significance Statement

Excitation/inhibition imbalance, which results in neural hyperexcitability, has been suggested to be involved in the etiology of autism. Both phasic and tonic inhibition play critical roles in the regulation of excitation/inhibition balance. Additionally, the postnatal maturation of inhibitory transmission controls the opening and closure of critical periods for cortical plasticity, a process underlying the formation of sensory perception, language acquisition and social development. We revealed that not only phasic inhibition but also tonic inhibitory transmission was reduced in a developmental-dependent fashion in a mouse model with deletion of the autism-risk gene, contactin-associated protein 2 (Cntnap2). Activation of δ-subunit containing tonic currents reduced neural excitability, indicating the agonist of δ-subunit containing GABA receptors could be a potential use in treatment of autism.

## Introduction

Autism spectrum disorder (ASD), characterized by qualitative differences in social communication, and restricted/repetitive behaviors, is a neurodevelopmental condition with heterogeneous phenotypes and diverse genetic association among individuals (Abrahams and Geschwind, 2008; State and Levitt, 2011). Genomics and gene expression studies have provided evidence supporting the hypothesis that altered GABAergic inhibition may be one of the common factors contributing to the emergence of the syndrome in ASD (Hussman, 2001; Rubenstein and Merzenich, 2003).

GABAergic inhibitory transmission is comprised of phasic inhibition and tonic inhibition. In the CNS, phasic inhibition is primarily mediated by GABA_A_ receptors (GABA_A_Rs) located at synaptic sites, while tonic inhibition involves GABA_A_Rs located perisynaptically and extrasynaptically at postsynaptic sites (Farrant and Nusser, 2005). The GABA_A_Rs contributing to these two types of inhibition possess distinct GABA binding affinities: GABA_A_Rs mediating tonic inhibition are activated by micromolar levels of ambient GABA and GABA spillover from synaptic clefts, while GABA_A_Rs involved in phasic inhibition are activated by synaptic GABA at millimolar levels (Glykys and Mody, 2007). In addition, activation of another type of GABA receptors, GABA_B_ receptors (GABA_B_Rs), can induce slow inhibitory synaptic currents via the stimulation of inwardly rectifying K^+^ channels (Heaney and Kinney, 2016).

Notably, GABAergic transmission plays an important role in the regulation of critical periods for brain plasticity (Gogolla et al., 2009), a process required for the refinement of sensory perception, language acquisition, and social development (Knudsen, 2004; Wang et al., 2014; Werker and Hensch, 2015). In visual cortex, the typical critical period in rodents occurs between postnatal weeks 3–5 in layer 2/3 (Hensch, 2005b). Disruption of GABAergic inhibition at different stages of development results in differentiated effects on the critical period. For example, in early development, GABAergic inhibition is required to initiate the critical period, whereas during later development it is involved in the termination of the critical period (Jiang et al., 2005).

Contactin-associated protein 2 (Cntnap2), which encodes a cell-adhesion molecule of the neurexin family (Arking et al., 2008), is an important autism-associated gene identified in previous studies (Alarcon et al., 2008; Hussman et al., 2011). Mice with knocked-out (KO) Cntnap2 (Cntnap2-KO) exhibit autism-related behaviors and seizure activity (Peñagarikano et al., 2011). It has been reported that deletion of Cntnap2 reduces the number of parvalbumin-positive interneurons in somatosensory cortex measured at postnatal day 14 (Peñagarikano et al., 2011) and disrupts hippocampal inhibition in adults (Jurgensen and Castillo, 2015). These lines of evidence suggested that GABAergic inhibition is abnormal in the brains of Cntnap2 KO mice. However, it remains elusive (1) whether both phasic and tonic inhibition are disrupted, and (2) when during postnatal development this disruption starts. In mice, Cntnap2 is expressed in multiple cortical sensory modalities, including visual cortex (Gordon et al., 2016). Visually evoked activity in dorsal stream associated visual areas of Cntnap2 KO mice is decreased (Townsend and Smith, 2017). Consistently, dysfunctions in visual sensory perception are frequently reported in individuals with autism (Bakroon and Lakshminarayanan, 2016).

In this study, we used the visual cortex as a model to investigate developmental changes in tonic and phasic inhibition in Cntnap2 KO mice. Our results show that phasic and tonic inhibition are disrupted in a developmentally-dependent manner, which provides significant insights into the potential timing of treatment for autism. Activation of δ-subunit-mediated tonic currents can reduce the excitability of pyramidal cells, suggesting that tonic inhibition could be a potential therapeutic target in autism.

## Materials and Methods

### Animals

Cntnap2^−/−^ mice (https://www.jax.org/strain/017482, stock number 017482, The Jackson Laboratory) were bred as heterozygous (HET) pairs, resulting in Cntnap2^+/+^ (wild type, WT), Cntnap2^−/−^ (KO), and Cntnap2^+/−^ (HET) offspring. Cntnap2 KO mice and WT littermate controls of either sex at postnatal three to four or six to eight weeks old were used. No significant sex effects were observed. Genotyping PCR was performed using the following primers: Caspr2-intron1 5’-TCA GAG TTG ATA CCC GAG CGC C-3’, G3M-Caspr2 5’-TGC TGC TGC CAG CCC AGA ACT GG-3’ and Caspr2-Neo 5’-TTG GGT GGA GAG GCT ATT CGG CTA TG-3’. PCRs were performed using GoTaq Green buffer with the following thermocycler parameters: 94°C for 1 min (94°C for 30 s, 66°C for 30 s, 72°C for 90 s) × 30 cycles, 72°C for 5 min. Animals were housed in the animal facility with free access to food and water, on a 12/12 h light/dark cycle. All animal procedures were performed in accordance with the Hussman Institute for Autism animal care committee’s regulations.

### Preparation of visual cortical slices

Mice were anesthetized by isoflurane inhalation before transcardial perfusion with ice-cold cutting buffer: 212.7 mM sucrose, 5 mM KCl, 1.25 mM NaH_2_PO_4_, 10 mM MgCl_2_, 0.5 mM CaCl_2_, 26 mM NaHCO_3_, and 10 mM dextrose, bubbled with 95% O_2_/5% CO_2_, pH 7.4. Brains were extracted and 300-µm coronal slices were cut on a vibrating microtome (Leica VT1200s) in the ice-cold cutting buffer. Slices containing visual cortex were transferred to a bath containing standard artificial CSF (ACSF): 124 mM NaCL, 5 mM KCl, 1.23 mM NaH_2_PO_4_, 26 mM NaHCO_3_, 10 mM dextrose, 1 mM MgCl_2_, and 2 mM CaCl_2_, and were incubated at 30°C for 30 min and then maintained at room temperature for at least an additional 30 min before recording.

### Visualized whole-cell patch-clamp recordings

Whole-cell recordings were made from visually-identified pyramidal cells in layer 2/3 of visual cortex using borosilicate glass microelectrodes (World Precision Instruments) pulled to a resistance of 3–7 MΩ filled with a Cs-based internal solution containing the following: 120 mM CsCl, 8 mM NaCl, 10 mM HEPES, 2 mM EGTA, 5 mM QX-314, 0.5 mM Na_2_GTP, 4 mM MgATP, and 10 mM Na_2_-phosphocreatine, pH adjusted to 7.3 with CsOH, 280–290 mOsm, or a K-based internal solution: 130 mM K-gluconate, 10 mM KCl, 10 mM HEPES, 0.2 mM EGTA, 4 mM MgATP, 0.5 mM Na_2_GTP, and 10 mM Na_2_-phosphocreatine, pH adjusted to 7.3 with KOH, 280–290 mOsm. Only pyramidal cells with an input resistance >75 MΩ and a series resistance <25 MΩ were studied. Whole-cell parameters were monitored throughout the recording with a 100-ms negative step (−6 mV for voltage-clamp mode and −40 pA for current-clamp mode, respectively) delivered every 30 s. Cells were excluded from analysis if series resistance changed >15% over the course of the experiment. Recordings were made with an Axon MultiClamp 700B amplifier (Molecular Devices). Data were filtered at 2 kHz and digitized at 10 kHz with a National Instruments digital-analog converter under the control of Igor Pro software (WaveMetrics, v6.37, RRID:SCR_000325).

### Measurement of tonic currents

Measurement of GABA_A_R-mediated tonic currents were conducted as previously described (Huang et al., 2015). Briefly, membrane currents were recorded in the presence of 10 µM NBQX and 100 µM DL-AP5 with the Cs-based internal solution in voltage-clamp mode (V_h_ = −70 mV). Tonic currents were measured as the shift in the holding currents following the addition of the GABA_A_R antagonist bicuculline methochloride (BMC; 20 µM) to ACSF containing 5 µM GABA. Holding currents were calculated by the generation of all-points histograms (bin width = 2.5 pA) for the GABAR-agonist and bicuculline epochs (30 s). The histograms were fitted with a Gaussian model, and only the left side of the distribution was used to avoid contamination with spontaneous IPSCs (sIPSCs). The difference between the peaks of these Gaussian fits was used as the value for the tonic inhibitory currents. Tonic currents were normalized to membrane capacitance (C_m_), to account for variability in cell size. C_m_ was calculated as the integral of transients induced by 100-ms test pulses. The same basic procedure was used to measure current shifts induced by the application of THIP (10 µM) and R-Baclofen (50 µM). For R-Baclofen recordings, the tonic current was calculated as the shift in holding current between the pre-drug baseline and R-Baclofen application; and recordings were made in voltage-clamp mode (V_h_ = −70 mV) using the K-based internal solution and an external ACSF with modified KCl (7 mM). Analysis of tonic currents was performed using custom scripts written in Igor Pro (WaveMetrics).

### Measurement of sIPSCs

The sIPSCs were recorded in voltage-clamp mode (V_h_ = −70 mV) from layer 2/3 pyramidal cells of visual cortical slices with the presence of 10 µM NBQX and 100 µM DL-AP5 to block glutamatergic synaptic transmissions. The Cs-based internal solution was used in these recordings. The frequency and amplitude of sIPSCs were analyzed by Mini Analysis software (Synaptosoft) as described previously (Gao et al., 2017). A threshold of 3× the RMS noise was used for event discrimination. At least 200 events per cell were used in these calculations. The rise and decay times were calculated using the average of 150–200 well-isolated events. The rise time was defined as the time interval between the start and the peak of the average trace. The decay time tau was obtained from one exponential fitting to the decay curve of the average trace.

### Measurement of intrinsic membrane excitability

Membrane potentials of layer 2/3 pyramidal cells in visual cortex were recorded in current-clamp mode with K-based internal solution. 10 µM NBQX, 100 µM DL-AP5, and 20 µM Gabazine were present in ACSF to block synaptic transmission. Current-clamp recordings were made at the resting membrane potential (RMP) of the cell. Membrane excitability was measured by injection of a 1000-ms ramp current or step currents of increasing amplitude (40-pA increments). Step current injection was used to determine the maximum firing rate, action potential features and interspike interval (ISI). The voltage threshold was defined as membrane potential where the first derivative (dV/dt) crossing a fixed criterion, 50 mV/ms (Sekerli et al., 2004; Kole and Stuart, 2008). The current threshold was defined as the level of injected current at the threshold. The action potential trough was defined as the minimum membrane potential between the peak and the next action potential, or the minimum membrane potential during 5 ms after the peak if only one action potential was induced. The action potential height was defined as the difference between the action potential peak and trough. The action potential width was defined as the width at half-height. The action potential upstroke was defined as the maximum value of dV/dt between the threshold and the peak, while the downstroke was defined as the minimum of dV/dt between the peak and the trough. First ISI and mean ISI were the ISI between the first two spikes and the mean value of all ISIs, respectively. Adaptation index was defined as: $\frac{1}{N-1}\overset{N-1}{\underset{n=1}{\sum }}\frac{{ISI}_{n+1}-{ISI}_{n}}{{ISI}_{n+1}+{ISI}_{n}}$, where *N* is the number of ISIs in the sweep. Firing rate was plotted as a function of input current.

To examine the effects of THIP on membrane excitability, layer 2/3 pyramidal cells were recorded with K-based internal solution in the presence of 10 µM NBQX and 100 µM DL-AP5. Step currents of increasing amplitude (40-pA increments, 500 ms) were used to evoke action potentials for determination of firing rate and current threshold. After a baseline recording, 10 µM THIP was added to the bath. After 5 min of superfusion with THIP, a second step-current recording was made. As above, firing rate was plotted as a function of input current. The initial slope of this curve (with *R* ^2^ ≥ 95.0%) was used as a measure of excitability, with comparisons made before and after THIP treatment within the same cell.

### Drugs/compounds

NBQX, BMC, THIP, GABA, Gabazine, and DL-AP5 were purchased from R&D Systems. R-Baclofen was purchased from Sigma-Aldrich. BMC, THIP, NBQX, GABA, Gabazine, and R-Baclofen were all dissolved in distilled water as stock solutions, then diluted 1:1000 in ACSF.

### Statistical analysis

All data are presented as mean ± SEM. Statistical analysis was performed using GraphPad Prism 6 software (GraphPad Prism, RRID:SCR_002798). All datasets were tested for normality using the D’Agostino-Pearson omnibus normality test. To determine the interaction between two factors (age and genotype), a two-way ANOVA was used. To compare the input-output curves in two conditions (before vs THIP, or WT vs KO), two-way repeated measures ANOVA was used. Two-group comparisons for unpaired experiments were made using two-tailed unpaired *t* tests, or Mann–Whitney *U* (M–W) tests if appropriate. For paired two-group comparisons, two-tailed paired *t* test or Wilcoxon matched-pairs signed rank test was used if appropriate. The threshold for significance was set at *p* = 0.05 (Table 1).

**Table 1.** Statistical table

	Description	Data structure	Type of test	Statistical value
a	sIPSC frequency (Fig. 1*B*)	Normal distribution	*t* test	*t*_(29)_ = 0.5388, *p* = 0.5941 (3–4 weeks); *t*_(29)_ = 2.116, *p* = 0.0431 (6–8 weeks)
b	sIPSC amplitude (Fig. 1*C*)	Normal distribution	*t* test; ANOVA	*t*_(29)_ = 0.5665, *p* = 0.5754 (3–4 weeks); *t*_(29)_ = 0.4330, *p* = 0.6682 (6–8 weeks); age: *F*_(1,58)_ = 20.27, *p* < 0.0001
c	sIPSC rise time (Fig. 1*E*)	Normal distribution	ANOVA	*F*_(1,60)_ = 2.188, *p* = 0.1443 (WT vs KO)
d	sIPSC decay time (Fig. 1*F*)	Normal distribution	ANOVA	*F*_(1,60)_ = 0.7872, *p* = 0.3785 (WT vs KO)
e	I_GABA_ in six to eight weeks (Fig. 2*B*)	Non-normal distribution	M–W test	*p* = 0.0282
f	Cm in I_GABA_ measurement	Normal distribution	*t* test	*t*_(36)_ = 0.4044, *p* = 0.6883
g	Normalized I_GABA_ in six to eight weeks (Fig. 2*C*)	Non-normal distribution	M–W test	*p* = 0.0058
h	I_GABA_ in three to four weeks (Fig. 2*B*)	Normal distribution	*t* test	*t*_(31)_ = 0.4277, *p* = 0.8637
i	Normalized I_GABA_ in three to four weeks (Fig. 2*C*)	Non-normal distribution	M–W test	*p* = 0.5529
j	C_m_ in I_THIP_ measurement	Normal distribution	*t* test	*t*_(45)_ = 0.1597, *p* = 0.8739
k	I_THIP_ (Fig. 3*B*)	Normal distribution	*t* test	*t*_(45)_ = 2.788, *p* = 0.0077
l	Normalized I_THIP_ (Fig. 3*C*)	Normal distribution	*t* test	*t*_(45)_ = 2.979, *p* = 0.0047
m	C_m_ in I_Baclofen_ measurement	Normal distribution	*t* test	*t*_(28)_ = 1.533, *p* = 0.1366
n	I_Baclofen_ (Fig. 4*B*)	Normal distribution	*t* test	*t*_(28)_ = 1.182, *p* = 0.247
o	Normalized I_Baclofen_ (Fig. 4*C*)	Non-normal distribution	M–W test	*p* = 0.9834
p	Ramp-current threshold (Fig. 5*A*)	Normal distribution	*t* test	*t*_(23)_ = 0.1924, *p* = 0.849 (3–4 weeks); *t*_(22)_ = 1.064, *p* = 0.299 (6–8 weeks)
q	Current-firing curve (Fig. 5*B*)		ANOVA	*F*_(1,23)_ = 0.0228, *p* = 0.881 (3–4 weeks); *F*_(1,22)_ = 1.320, *p* = 0.263, (6–8 weeks)
r	Step-current threshold (Fig. 5*C*)	Normal distribution	*t* test	*t*_(23)_ = 0.2003, *p* = 0.843 (3–4 weeks); *t*_(22)_ = 0.8177, *p* = 0.4223 (6–8 weeks)
s	Max. firing rate (Fig. 5*D*)	Normal distribution	*t* test	*t*_(23)_ = 0.69, *p* = 0.4971 (3–4 weeks); *t*_(22)_ = 0.9258, *p* = 0.3646 (6–8 weeks)
t	Current-firing curve in three to four weeks (Fig. 6*B*)		ANOVA	*F*_(1,24)_ = 28.45, *p* < 0.0001 (WT, before vs THIP); *F*_(1,24)_ = 14.66, *p* = 0.0008 (KO, before vs THIP); *F*_(1,24)_ = 1.199, *p* = 0.2844 (before, WT vs KO); *F*_(1,24)_ = 0.0073, *p* = 0.9328 (THIP, WT vs KO)
u	Current-firing curve in six to eight weeks (Fig. 6*C*)		ANOVA	*F*_(1,30)_ = 57.87, *p* < 0.0001 (WT, before vs THIP); *F*_(1,32)_ = 30.66, *p* = 0.0008 (KO, before vs THIP); *F*_(1,31)_ = 1.228, *p* = 0.2764 (before, WT vs KO); *F*_(1,31)_ = 4.919, *p* = 0.0340 (THIP, WT vs KO)
v	Step-current threshold (Fig. 6*D*)	Normal distribution Non-normal distribution	Paired *t* test Wilcoxon test	*t*_(12)_ = 7.229, *p* < 0.0001 (WT); *p* = 0.0005(KO)
w	Step-current threshold (Fig. 6*E*)	Non-normal distribution Normal distribution	Wilcoxon test Paired *t* test	*p* < 0.0001 (WT); *t*_(16)_ = 6.154, *p* < 0.0001 (KO)
x	Δ current threshold (Fig. 6*F*)	Non-normal distribution	M–W test	*p* = 0.0257
y	Slope in three to four weeks (Fig. 6*G*)	Non-normal distribution	Wilcoxon test	*p* = 0.0002 (WT) *p* = 0.0002 (KO)
z	Slope in six to eight weeks (Fig. 6*H*)	Normal distribution Non-normal distribution	Paired *t* test Wilcoxon test	*t*_(15)_ = 5.761, *p* < 0.0001 (WT); *p* = 0.0001 (KO)
aa	Δ slope (Fig. 6*I*)	Non-normal distribution	M–W test	*p* = 0.0063

## Results

### Phasic inhibition in Cntnap2 KO mice is altered in an age-dependent fashion

**Figure 1.**
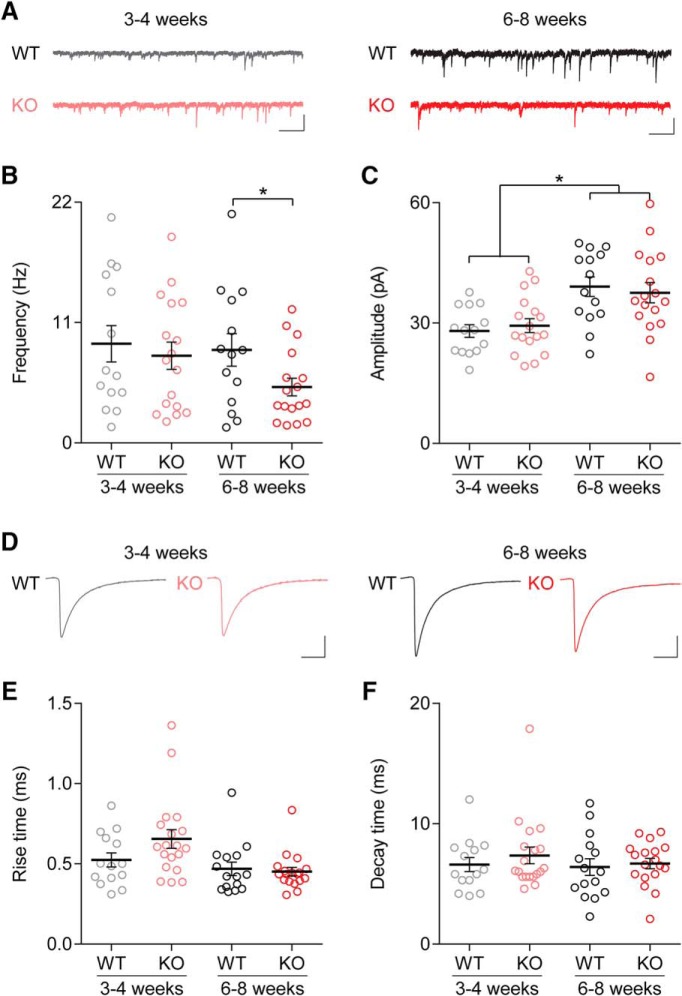
Phasic inhibition is altered in Cntnap2 KO mice in an age-dependent manner. ***A***, Example traces of sIPSC activity recorded from layer 2/3 pyramidal cells in WT (upper traces) and KO (lower traces) mice at three to four weeks old (left) and six to eight weeks old (right). ***B***, The frequency was lower in KO mice compared to WT at six to eight weeks. There was no effect of age on the frequency of sIPSCs, and no statistical difference between the three- to four-week-old KO and WT groups. ***C***, The amplitude of sIPSCs was significantly increased during postnatal development, but there was no effect of genotype. ***D***, Average sIPSC waveforms for Cntnap2 WT and KO mice in both the three- to four- and six- to eight-week age groups. ***E***, There was no significant effect of genotype or age group on the sIPSC rise time. ***F***, There was no significant effect of genotype or age group on the sIPSC decay time tau. Scale bars: 50 pA, 0.5 s (***A***); 10 pA, 100 ms (***D***); *p < 0.05. Error bars show SEM.

During postnatal development, inhibition has differential roles in controlling the formation of critical periods for brain plasticity. For example, in visual cortex the normal development of GABAergic inhibition during postnatal week 3 is required to open the critical period, and the maturation of inhibitory innervation after postnatal week 5 is involved in closing the critical period (Hensch, 2005b; Jiang et al., 2005; Levelt and Hübener, 2012). A previous study in Cntnap2 KO mice reported that phasic inhibition is altered in adult hippocampus (Jurgensen and Castillo, 2015); however, when this disruption takes place is not clear. Therefore, we examined phasic inhibition in Cntnap2 KO mice during postnatal development. We recorded spontaneous inhibitory postsynaptic currents (sIPSCs) from layer 2/3 pyramidal cells in visual cortex of Cntnap2 KO mice and WT controls at two postnatal ages, three to four weeks (P21-P28) and six to eight weeks (P42-P56) old. sIPSCs were recorded as inward currents by using a high Cl^−^ internal solution and holding at negative potentials (Fig. 1*A*). We found that in the absence of Cntnap2, the frequency of sIPSCs was significantly reduced in mice aged six to eight weeks (WT: 8.50 ± 1.48 Hz, *n* = 14 cells/five animals; KO: 5.10 ± 0.80 Hz, *n* = 17 cells/five animals; *t*_(29)_ = 2.116, *p* = 0.0431^a^, *t* test; Fig. 1*B*), while there was no significant effect of genotype on sIPSC frequency at three to four weeks (WT: 9.059 ± 1.659 Hz, *n* = 14/3; KO: 7.96 ± 1.25 Hz, *n* = 17/5; *t*_(29)_ = 0.5388, *p* = 0.5941^a^, *t* test; Fig. 1*B*). The amplitude of sIPSCs was not altered, compared to WT mice, by the deletion of Cntnap2 at either three to four or six to eight weeks, although amplitude increased significantly during development in both KO and WT mice (three to four weeks, WT: 28.72 ± 2.16 pA, *n* = 14/3; KO: 29.89 ± 1.74 pA, *n* = 17/5; *t*_(29)_ = 0.5665, *p* = 0.5754^b^, *t* test; six to eight weeks, WT: 39.07 ± 2.42 pA, *n* = 14/5, KO: 37.53 ± 2.53 pA, *n* = 17/5; *t*_(29)_ = 0.4330, *p* = 0.6682^b^, *t* test; age: *F*_(1,58)_ = 20.27, *p* < 0.0001^b^, ANOVA; Fig. 1*C*). We further analyzed the kinetics of sIPSCs (average waveforms, Fig. 1*D*), reflecting the composition and phosphorylation of GABA_A_R subunits mediated in phasic inhibition (Labrakakis et al., 2014), and found that genotype had no effect on the rise time or the decay time of sIPSCs (rise time: three to four weeks, WT: 0.52 ± 0.04 ms, KO: 0.66 ± 0.06 ms; six to eight weeks, WT: 0.47 ± 0.05 ms, KO: 0.45 ± 0.03 ms; *F*_(1,60)_ = 2.188, *p* = 0.1443^c^, ANOVA, Fig. 1*E*; decay time tau: three to four weeks, WT: 6.61 ± 0.58 ms, KO: 7.36 ± 0.69 ms; six to eight weeks, WT: 6.40 ± 0.69ms, KO: 6.69 ± 0.43 ms; *F*_(1,60)_ = 0.7872, *p* = 0.3785^d^, ANOVA, Fig. 1*F*). Collectively, these data are suggestive of a developmental role for Cntnap2 in the maturation and stability of GABAergic synapses in visual cortex.

### GABA_A_R-mediated tonic inhibition is reduced in Cntnap2 KO mice in an age-dependent manner

Ambient GABA or GABA spillover from synaptic clefts activates tonic inhibitory conductance, which is largely mediated by extrasynaptic and perisynaptic GABA_A_Rs (Farrant and Nusser, 2005). In acute brain slices, the ambient GABA level may vary with many factors, such as the depth of the neuron in the slice, and the level of local GABA release and uptake. To standardize the ambient GABA levels around the recorded neurons, we bath applied GABA at a concentration of 5 µM. This concentration of GABA was previously shown to activate extrasynaptic/perisynaptic GABARs without activating lower-affinity synaptic receptors, and to maintain ambient GABA close to the *in vivo* level (Glykys and Mody, 2006, 2007; Bright and Smart, 2013). Tonic inhibitory conductance was estimated as the difference in the holding currents during bath application of GABA (5 µM) followed by coapplication of 20 µM BMC (Bright and Smart, 2013; Huang et al., 2015).

**Figure 2.**
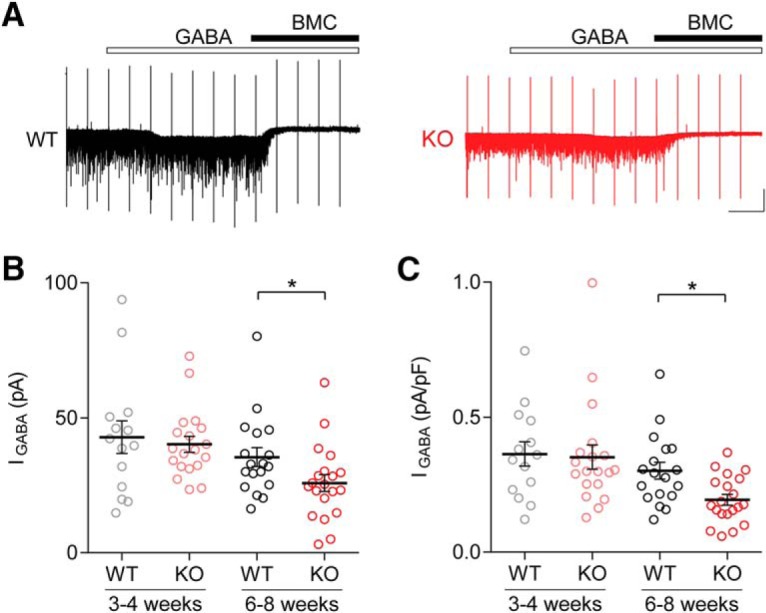
GABA-induced tonic inhibitory currents are reduced in Cntnap2 KO mice in an age-dependent fashion. ***A***, Example traces showing tonic inhibitory currents induced by 5 µM GABA in six- to eight-week-old WT (left trace) and KO (right trace) animals. Peaks are capacitive transients in response to voltage steps used to monitor series resistance. ***B***, ***C***, In slices from three- to four-week-old animals, there was no effect of *Cntnap2* KO on pyramidal cell tonic current (I_GABA_) amplitude (***B***), or normalized tonic current density (***C***). In slices from six- to eight-week-old animals, KO pyramidal cells exhibited reduced tonic current amplitude (***B***), and reduced tonic current density (***C***) compared to cells from WT mice. Scale bars: 100 pA, 50 s (***A***); *p < 0.05. Error bars show SEM.

Accordingly, we performed these experiments in Cntnap2 WT and Cntnap2 KO mice at two different ages; three to four and six to eight weeks. As shown in Figure 2, the results in six- to eight-week-old KO and WT mice indicated a significant difference in tonic inhibitory conductance. The amplitude of the tonic current was significantly affected by genotype (WT: 35.48 ± 3.53 pA, *n* = 18 cells/five animals; KO: 25.82 ± 3.10 pA, *n* = 20 cells/five animals; *p* = 0.0282^e^, M–W test; Fig. 2*B*). To account for the effect of cell size, we normalized the tonic currents by cell membrane capacitance, which was not different between KO and WT (WT: 125.8 ± 9.22 pF, *n* = 18/5; KO: 131.0 ± 8.79 pF, *n* = 20/5; *t*_(36)_ = 0.4044, *p* = 0.6883^f^, *t* test). The results shown in Figure 2 indicated that the normalized tonic current is significantly reduced in KO mice at this age (WT: 0.30 ± 0.03 pA/pF; KO: 0.19 ± 0.02 pA/pF; M–W test, *p* = 0.0058^g^; Fig. 2*C*). Similar to the developmental effect of Cntnap2 KO on phasic inhibition, no difference in tonic inhibition was found in three- to four-week-old KO and WT mice (WT: 42.91 ± 6.04 pA, *n* = 14/3; KO: 40.26 ± 2.95 pA, *n* = 19/5; *t*_(31)_ = 0.4277, *p* = 0.8637^h^, *t* test; Fig. 2*B*), or in the tonic currents normalized by cell capacitance (WT: 0.36 ± 0.05 pA/pF; KO, 0.35 ± 0.05 pA/pF; M–W test, *p* = 0.5529^i^; Fig. 2*C*). Taken together, these results indicate that the deletion of Cntnap2 leads to reduced tonic inhibitory conductance in cortical pyramidal cells in a developmentally-dependent manner.

**Figure 3.**
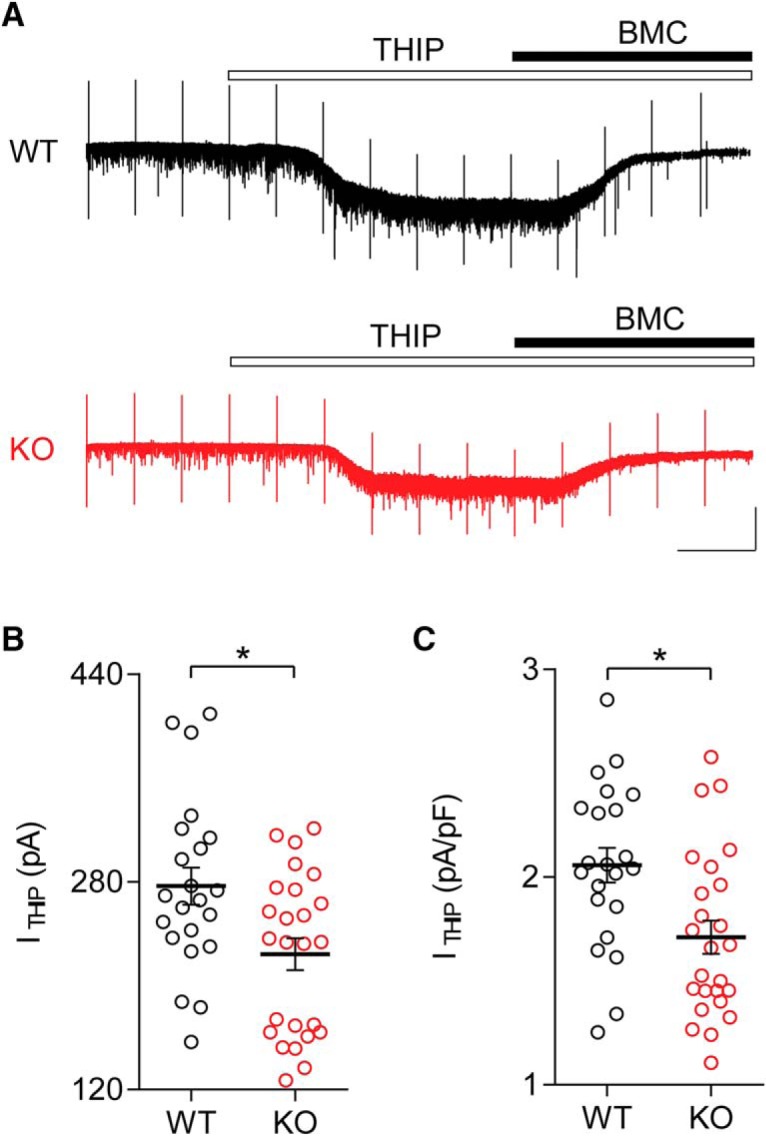
Reduction of δ-subunit-mediated tonic currents in six- to eight-week-old Cntnap2 KO mice. ***A***, Example traces from layer 2/3 pyramidal cells from six- to eight-week-old WT (top) and KO (bottom) mice showing the current induced by exposure to 10 µM THIP, an agonist of δ-subunit-containing GABA_A_Rs. Peaks are capacitive transients in response to voltage steps used to monitor series resistance. ***B***, ***C***, Cells from KO animals exhibited smaller THIP-induced tonic currents (I_THIP_; ***B***) and smaller normalized tonic current density (***C***). Scale bars: 100 pA, 50 s (***A***); *p < 0.05. Error bars show SEM.

In pyramidal cells of cortical layer 2/3, tonic inhibition is mediated by extrasynaptic GABA_A_Rs containing the δ-subunit. THIP is a potent activator of GABARs which contain the δ-subunit, and its bath application at 10 µM induces a large tonic current in layer 2/3 pyramidal cells in visual cortex (Huang et al., 2015). Based on the reduction in tonic inhibitory conductance observed in six- to eight-week-old Cntnap2 KO mice, we hypothesized that pyramidal cells from mutant mice could exhibit a reduced tonic inhibitory current mediated specifically by GABARs with the δ-subunit. We used bath application of 10 µM THIP followed by coapplication of 20 µM BMC to quantify this current in Cntnap2 KO and WT mice (Fig. 3*A*). As we observed previously, C_m_ was not affected by genotype (WT: 136.0 ± 6.1 pF, *n* = 22 cells/five animals; KO: 134.4 ± 7.64 pF, *n* = 25 cells/six animals; *t*_(45)_ = 0.1597, *p* = 0.8739^j^, *t* test), but the THIP-induced tonic current (WT: 276.8 ± 14.4 pA, *n* = 22 cells/five animals; KO: 224.4 ± 12.3 pA, *n* = 25 cells/six animals; *t*_(45)_ = 2.788, *p* = 0.0077^k^, *t* test; Fig. 3*B*) and the tonic current density (WT: 2.06 ± 0.08 pA/pF, *n* = 22 cells/five animals; KO: 1.71 ± 0.08 pA/pF, *n* = 25 cells/six animals; *t*_(45)_ = 2.979, *p* = 0.0047^l^, *t* test; Fig. 3*C*) were both significantly smaller in KO mice than in their WT counterparts, which confirms that δ-subunit meditated GABA_A_Rs are disrupted in six- to eight-week-old Cntnap2-KO mice.

### GABA_B_R-mediated tonic inhibition is not affected in Cntnap2 KO mice

**Figure 4.**
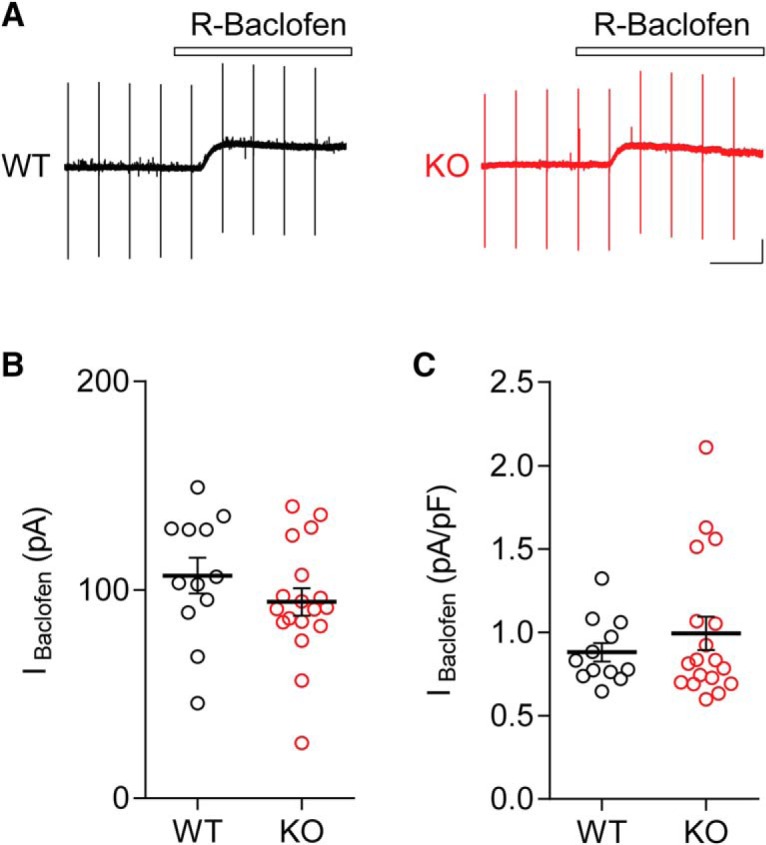
GABA_B_R-mediated slow inhibitory currents do not change in Cntnap2 KO mice. ***A***, Example traces from layer 2/3 pyramidal cells from six- to eight-week-old WT (left) and KO (right) mice, showing the current induced by exposure to 100 µM R-Baclofen, an activator of GABA_B_Rs. Peaks are capacitive transients in response to voltage steps used to monitor series resistance. ***B***, ***C***, No effect of genotype was found on R-Baclofen-induced current (I_Baclofen_) amplitude (***B***), or normalized current density (***C***). Scale bars: 100 pA, 50 s (***A***). Error bars show SEM.

Ambient GABA can activate metabotropic GABA_B_Rs, which are another class of extrasynaptically-located GABARs (Scanziani, 2000; Kulik et al., 2003). While GABA_A_Rs are Cl^-^ passing ion channels, the GABA_B_Rs are functionally-distinct metabotropic receptors that exert their inhibitory effects through the downstream activation of K^+^ channels (Wu et al., 2011). Recently, there has been interest, both basic and clinical, in the potential use of R-Baclofen as a therapeutic agent for the amelioration of some symptoms of autism spectrum conditions (Berry-Kravis et al., 2012; Qin et al., 2015; Silverman et al., 2015). Because of the reduced GABA- and THIP-induced tonic currents we observed in Cntnap2 KO mice, it was intriguing to investigate the possibility that GABA_B_R-mediated currents could also be impacted by the loss of Cntnap2. R-Baclofen induced currents were recorded in pyramidal cells in layer 2/3 of visual cortical slices from six- to eight-week-old mice. As in previous experiments, we did not observe any effect of genotype on C_m_ (WT: 122.9 ± 9.55 pF, *n* = 12 cells/three animals; KO: 103.0 ± 8.52, *n* = 18 cells/four animals; *t*_(28)_ = 1.533, *p* = 0.1366^m^, *t* test). Interestingly, there was no statistical difference in the amplitude of R-Baclofen-induced currents (WT: 107.00 ± 8.64 pA, *n* = 12 cells/three animals; KO: 94.37 ± 6.55 pA, *n* = 18 cells/four animals; *t*_(28)_ = 1.182, *p* = 0.247^n^, *t* test; Fig. 4*B*), or normalized current density (WT: 0.88 ± 0.06 pA/pF, *n* = 12 cells/three animals; KO: 1.00 ± 0.10, *n* = 18 cells/four animals; M-W test, *p* = 0.9834^°^; Fig. 4*C*) in Cntnap2 KO mice compared to controls.

### Unchanged intrinsic excitability in Cntnap2 KO mice

**Figure 5.**
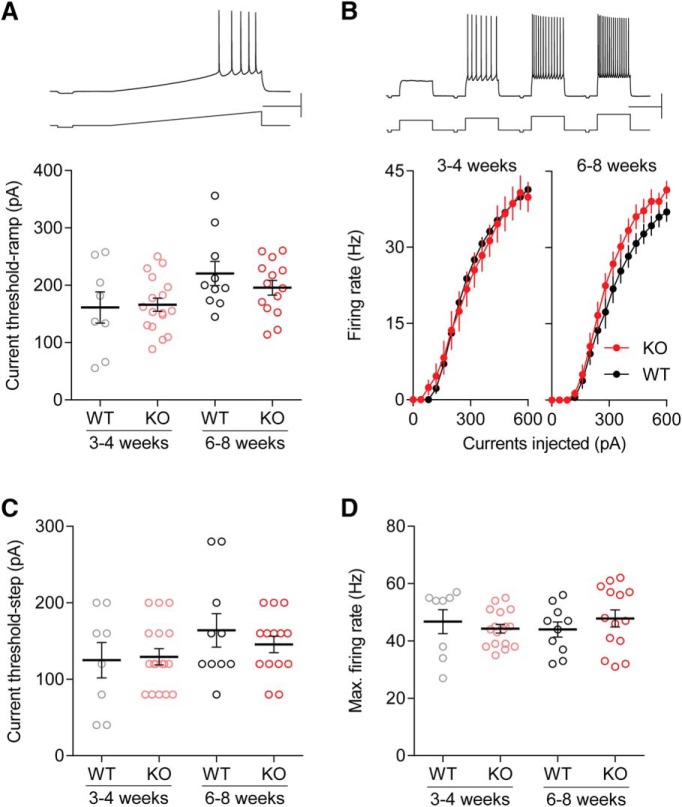
Intrinsic excitability in layer 2/3 pyramidal cells. ***A***, Membrane excitability was tested by injection of a ramp current. An example trace is shown at top. No effect of genotype or age was found in the current threshold in ramp tests. ***B***, The injection of step currents of increasing amplitude was used for the determination of membrane excitability. There was no significant difference in the function of firing rate versus input current between WT and KO mice in either age groups (3–4 or 6–8 weeks). ***C***, Step current injection revealed no effect of genotype or age on the current threshold. ***D***, Step current injection revealed no effect of genotype or age on the maximum firing rate. Scale bars: 20 mV, 250 pA, 1 s (***A***); 20 mV, 250 pA, 0.25 s (***B***). Error bars show SEM.

Cntnap2 has been reported to regulate axonal excitability by clustering voltage-gated potassium channels at the juxtaparanodes of myelinated axons (Poliak et al., 2003). Deletion of Cntnap2 might influence this neural excitability. Therefore, we conducted a series of experiments to assess the intrinsic excitability and action potential properties of layer 2/3 pyramidal cells in visual cortex of Cntnap2 KO and WT mice at both three to four and six to eight weeks old, to determine if potential differences in excitability are attributable solely to GABAergic input or include cell-autonomous factors (Fig. 5). We found that there was no effect of genotype on resting membrane potential (RMP) or input resistance in either of the age groups (Table 2). Using tests with ramp current injection, we did not find any significant difference in the current threshold between KO and WT in either of the age groups tested (three to four weeks, WT: 165.9 ± 11.97 pA, *n* = 17 cells; KO: 161.1 ± 27.29, *n* = 8 cells, *t*_(23)_ = 0.1924, *p* = 0.849^p^, *t* test; six to eight weeks, WT: 220.42 ± 21.01 pA, *n* = 10 cells; KO: 195.57 ± 12.78 pA, *n* = 14 cells, *t*_(22)_ = 1.064, *p* = 0.299^p^, *t* test; Fig. 5*A*). We also used step current injection to test current thresholds and firing frequency. Genotype did not have a significant effect on the relationship between firing rate and the injected current in three- to four-week-old mice (WT: *n* = 17; KO: *n* = 8; *F*_(1,23)_ = 0.0228, *p* = 0.881^q^, ANOVA; Fig. 5*B*). In six- to eight-week-old mice, the firing rate versus input current curve in KO mice was slightly shifted to the left compared to that of WT mice, but the difference was not statistically significant (WT: *n* = 10; KO: *n* = 14; *F*_(1,22)_ = 1.320, *p* = 0.263^q^, ANOVA; Fig. 5*B*). Step-current threshold (WT: 129.4 ± 10.91 pA, *n* = 17; KO: 125.0 ± 23.22, *n* = 8; *t*_(23)_ = 0.2003, *p* = 0.843^r^, *t* test; Fig. 5*C*) and maximum firing rate (WT: 44.3 ± 1.50 Hz, *n* = 17; KO: 46.8 ± 4.17, *n* = 8; *t*_(23)_ = 0.69, *p* = 0.4971^s^, *t* test; Fig. 5*D*) were similarly unaffected in three- to four-week-old mice. Likewise, in six- to eight-week-old mice, there was no effect of genotype on step current threshold (WT: 164 ± 21.87 pA, *n* = 10; KO: 145.71 ± 10.78, *n* = 14; *t*_(22)_ = 0.8177, *p* = 0.4223^r^, *t* test; Fig. 5*C*) or maximum firing rate (WT: 44 ± 2.64 Hz, *n* = 10; KO: 47.86 ± 2.96 Hz, *n* = 14; *t*_(22)_ = 0.9258, *p* = 0.3646^s^, *t* tes*t*; Fig. 5*D*). We further analyzed action potential features in three- to four- and six- to eight-week-old mice. As shown in the Table 2, there was no significant change in spikes features in pyramidal cells by the deletion of Cntnap2, except for a larger upstroke/downstroke ratio and smaller first ISI in six- to eight-week KO mice. Taken together, these results indicate that deletion of Cntnap2 does not affect the intrinsic excitability nor most of the spike features of pyramidal cells in visual cortex during postnatal development.

**Table 2.** Membrane properties and spike features in pyramidal cells of layer 2/3 visual cortex

	Three to four weeks			Six to eight weeks		
	WT	KO	Statistical value	WT	KO	Statistical value
Input resistance (MΩ)	91.51 ± 4.96	104.33 ± 13.07	*t*_(23)_ = 1.125 *p* = 0.2721	75.49 ± 6.19	82.32 ± 5.57	*t*_(22)_ = 0.8119 *p* = 0.4255
V_spike peak (mV)	46.41 ± 1.17	42.93 ± 2.65	*t*_(23)_ = 1.407 *p* = 0.1727	48.54 ± 1.65	46.75 ± 1.34	*t*_(22)_ = 0.8498 *p* = 0.4046
V_trough (mV)	−47.87 ± 0.44	−49.13 ± 0.94	*t*_(23)_ = 1.388 *p* = 0.1785	−48.08 ± 0.59	−48.46 ± 0.62	*t*_(22)_ = 0.4247 *p* = 0.6752
Spike height (mV)	94.28 ± 1.41	92.06 ± 3.15	*t*_(23)_ = 0.7528 *p* = 0.4592	96.63 ± 1.97	95.21 ± 1.62	*p* = 0.5036*
Spike width (ms)	1.18 ± 0.04	1.22 ± 0.05	*t*_(23)_ = 0.5595 *p* = 0.5812	1.05 ± 0.06	0.92 ± 0.03	*t*_(22)_ = 2.069 *p* = 0.0505
V_threshold (mV)	−37.25 ± 0.56	−38.67 ± 0.90	*t*_(23)_ = 1.401 *p* = 0.1747	−38.20 ± 1.02	−39.14 ± 0.58	*t*_(22)_ = 0.8592 *p* = 0.3995
Upstroke (mV/ms)	265.83 ± 8.82	250.42 ± 18.31	*t*_(23)_ = 0.8626 *p* = 0.3973	349.53 ± 17.73	358.53 ± 12.56	*p* = 0.9431*
Downstroke (mV/ms)	−68.02 ± 2.31	−65.86 ± 3.44	*t*_(23)_ = 0.5238 *p* = 0.6054	−79.90 ± 4.59	−91.96 ± 3.79	*t*_(22)_ = 2.033 *p* = 0.0543
Upstroke/downstroke ratio	−3.93 ± 0.11	−3.79 ± 0.18	*t*_(23)_ = 0.7090 *p* = 0.4854	−4.45 ± 0.26	−3.93 ± 0.11	*t*_(22)_ = 2.088***p*** **= 0.0486**
RMP (mV)	−68.40 ± 0.81	−67.01 ± 1.71	*p* = 0.6659*	−70.27 ± 1.10	−69.72 ± 0.83	*t*_(22)_ = 0.4048 *p* = 0.6895
First ISI (ms)	6.95 ± 0.26	7.86 ± 1.47	*t*_(23)_ = 0.8638 *p* = 0.3966	6.41 ± 0.46	5.26 ± 0.58	****p****= 0.0220*
Mean ISI (ms)	26.99 ± 0.76	28.63 ± 2.24	*p* = 0.7504*	29.41 ± 1.43	26.41 ± 1.44	*t*_(22)_ = 1.436 *p* = 0.1650
Adaptation index	0.02 ± 0.001	0.02 ± 0.001	*t*_(23)_ = 0.6344 *p* = 0.5321	0.02 ± 0.004	0.03 ± 0.002	*p* = 0.7879*
Slope of current-firing curve	7.02 ± 0.23	6.41 ± 0.60	*p* = 0.0883*	6.35 ± 0.28	7.19 ± 0.47	*p* = 0.1583*

Spike features were obtained from the first action potential evoked by the step current which was just above the threshold. ISIs were obtained from the response evoked by the step current which was 360 pA above the threshold. Two-tailed unpaired *t* test or M–W test (*) was used for statistical analyses. Bold text indicates statistical significance, *p* < 0.05.

### Activation of δ-subunit-containing GABA_A_Rs reduces neuronal excitability

**Figure 6.**
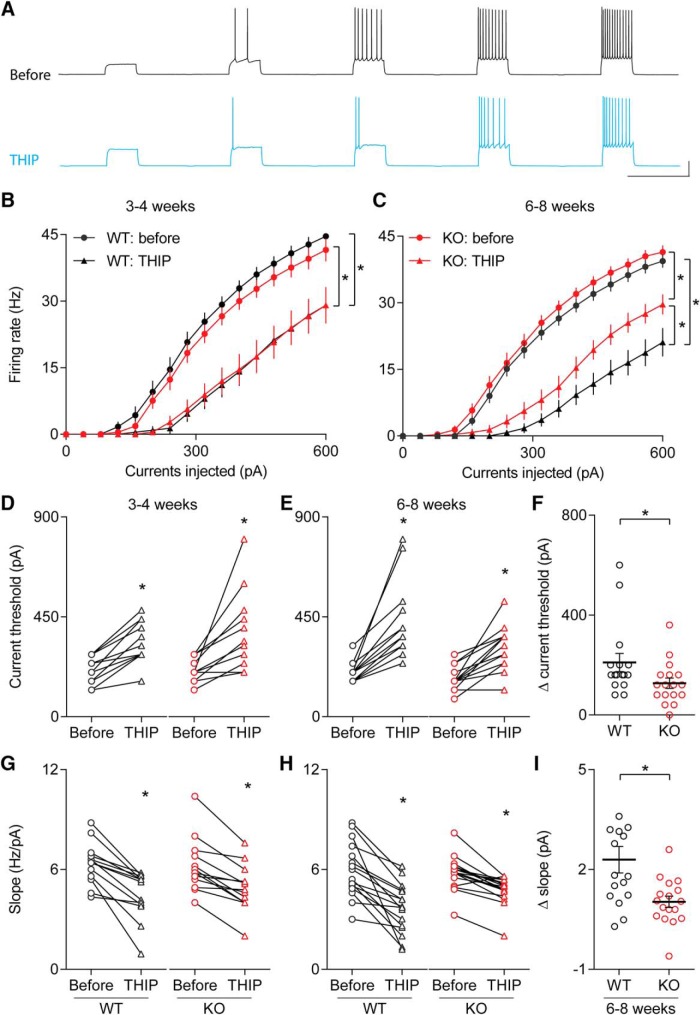
Application of THIP, the δ-subunit containing GABA_A_R agonist, reduces pyramidal cell excitability. ***A***, Example traces of action potential firing induced by step-current injection before and after bath application of THIP. Step currents were applied to layer 2/3 pyramidal cells to probe action potential firing frequency before and after the application of 10 µM THIP. ***B***, Graphical comparison of the input current versus firing rate relationship in three- to four-week-old WT and KO mice, before and after exposure to 10 µM THIP. We observed a main effect of drug treatment exposure on the input-output curve, but no effect of genotype and no treatment × genotype interaction. ***C***, Graphical comparison of the input current versus firing rate relationship in six- to eight-week-old WT and KO mice, before and after exposure to 10 µM THIP. The increase in the current threshold of action potential induction was significantly higher in WT than in KO cells. ***D***, Bath application of 10 µM THIP significantly increased the current threshold in both WT and KO mice at three to four weeks old. ***E***, Bath application of 10 µM THIP significantly increased the current threshold of spike induction in both WT and KO mice at six to eight weeks old. ***F***, Comparison of the change in current threshold of spike induction before and after THIP application in six- to eight-week-old Cntnap2 WT and KO littermates. ***G***, Superfusion with 10 µM THIP significantly reduced the slope of current-firing curve in three- to four-week-old WT and KO mice. ***H***, Superfusion with 10 µM THIP significantly reduced the slope of current-firing curve in six- to eight-week-old WT and KO mice. ***I***, Comparison of the change in the initial firing-rate slope (Δ-slope) before and after THIP application in six- to eight-week-old Cntnap2 WT and KO littermates. The reduction in the slope was significantly lower in KO mice than in WT. Scale bars: 20 pA, 1 s (***A***); *p < 0.05. Error bars show SEM.

Since neural hyperactivity is extensively implicated in autism, we next quantified the effects of tonic GABA_A_R activation on neuronal excitability in visual cortex using whole-cell current-clamp recordings from layer 2/3 pyramidal cells in WT and KO mice at both three to four and six to eight weeks of age. Step current injections of increasing amplitude were used to induce action potentials (Fig. 6*A*). We examined the relationship of firing rate versus input currents, the current threshold, and the slope of the input-output curves both before and during bath application of 10 µM THIP. In three- to four-week-old animals, the relationship of firing rate versus input current revealed a significant effect of THIP treatment in both WT and KO mice (before vs THIP, WT: *F*_(1,24)_ = 28.45, *p* < 0.0001^t^; KO: *F*_(1,24)_ = 14.66, *p* = 0.0008^t^; ANOVA; Fig. 6*B*), but no effect of genotype either before or during THIP application (WT vs KO, before: *F*_(1,24)_ = 1.199, *p* = 0.2844^t^; THIP: *F*_(1,24)_ = 0.0073, *p* = 0.9328^t^; ANOVA; Fig. 6*B*). In this age group, THIP significantly elevated the current threshold for action potential induction compared to the baseline in both WT and KO mice (WT, *n* = 13 cells, before: 203.08 ± 25.78 pA, THIP: 329.23 ± 40.10 pA; *t*_(12)_ = 7.229, *p* < 0.0001^v^, paired *t* test; KO, *n* = 13 cells, before: 209.23 ± 22.10 pA, THIP: 376.32 ± 60.54 pA; *p* = 0.0005^v^, Wilcoxon signed rank test; Fig. 6*D*), while the initial slope of the firing rate versus current step curve was reduced by application of THIP (WT, *n* = 13 cells, before: 6.36 ± 0.35 Hz/pA, THIP: 4.45 ± 0.40 Hz/pA; *p* = 0.0002^y^, Wilcoxon test; KO, *n* = 13 cells, before: 6.22 ± 0.45 Hz/pA, THIP: 4.89 ± 0.39 Hz/pA; *p* = 0.0002^y^, Wilcoxon test; Fig. 6*G*).

We also tested the effects of THIP application in six- to eight-week-old mice. Because of the deficits in tonic inhibitory conductance we observed in this age group, we hypothesized that the effects of δ-subunit activation by THIP on neuronal excitability would be attenuated by the loss of Cntnap2. A comparison of the curves of input current versus firing rate found a significant main effect of THIP application in both WT and KO mice (before vs THIP, WT: *F*_(1,30)_ = 57.87, *p* < 0.0001^u^; KO: *F*_(1,32)_ = 30.66, *p* = 0.0008^u^; ANOVA; Fig. 6*C*), with a significant genotype effect during THIP application, but not at baseline (WT vs KO, before: *F*_(1,31)_ = 1.228, *p* = 0.2764^u^; THIP: *F*_(1,31)_ = 4.919, *p* = 0.0340^u^; ANOVA; Fig. 6*C*). Exposure to THIP increased the current threshold in both genotypes (WT, *n* = 16 cells, before: 195.00 ± 10.88 pA, THIP: 405.00 ± 41.13 pA; *p* < 0.0001^w^, Wilcoxon test; KO, *n* = 17 cells, before: 181.18 ± 13.77 pA, THIP: 308.24 ± 22.69 pA; *t*_(16)_ = 6.154, *p* < 0.0001^w^, paired *t* test; Fig. 6*E*), but the average change in the threshold current was significantly lower in KO than in WT cells (Δ-step threshold; WT: 210.00 ± 36.42, *n* = 16 cells; KO: 127.06 ± 20.65 pA, *n* = 17 cells; *p* = 0.0257^x^, M–W test; Fig. 6*F*). Similarly, while THIP decreased the firing rate versus current step slope in both genotypes (WT, *n* = 16 cells, before: 5.96 ± 0.41 Hz/pA, THIP: 3.67 ± 0.37 Hz/pA; *t*_(15)_ = 5.761, *p* < 0.0001^z^, paired *t* test; KO, *n* = 17 cells, before: 5.79 ± 0.28 Hz/pA, THIP: 4.76 ± 0.23 Hz/pA; *p* = 0.0001^z^, Wilcoxon test; Fig. 6*H*), this effect was diminished in Cntnap2 KO mice compared to WT littermates (Δ-slope, WT: 2.30 ± 0.40, *n* = 16 cells; KO: 1.03 ± 0.19, *n* = 17 cells; *p* = 0.0063^aa^, M–W test; Fig. 6*I*). These data suggested that the activation of GABA_A_Rs on layer 2/3 pyramidal neurons has a profound effect on reducing neuronal excitability, which could have implications for reducing neural hyperactivity implicated in autism.

## Discussion

In the present study, we demonstrated an age-dependent effect of Cntnap2 deletion on both tonic and phasic inhibition in layer 2/3 pyramidal cells in the visual cortex. In Cntnap2 KO mice at three to four weeks old, we did not detect any changes in GABA-induced tonic inhibitory currents or in the amplitude or frequency of fast inhibitory synaptic transmission. In older animals, aged six to eight weeks, the deletion of Cntnap2 led to a reduction in GABA_A_R-mediated tonic inhibitory currents and reduced frequency of fast synaptic inhibitory events. Furthermore, the reduction in tonic inhibitory conductance was associated with reduction of δ-subunit containing GABA_A_R currents, with no reduction in GABA_B_R currents. Activation of δ-subunit-mediated tonic currents reduced the excitability of pyramidal cells, and this effect was smaller in six- to eight-week-old KO mice compared to WT.

Our findings indicate a developmental role of *Cntnap2* in the formation and maintenance of cortical GABAergic synapses, due to the age-dependence of the inhibitory deficits we discovered in these animals. From puberty to adulthood, inhibitory transmission increases gradually (Huang et al., 1999; Jiang et al., 2010). The maturation of inhibition plays an important role in the initiation and termination of the critical period for cortical plasticity (Hensch, 2005a; Jiang et al., 2005). In the mouse visual cortex, the initiation of the critical period at three weeks postnatal requires minimal inhibition, whereas the termination of the typical critical period at five weeks postnatal involves full maturation of inhibitory transmission. Our data show that Cntnap2 KO mice exhibit reduced phasic and tonic inhibition at six to eight weeks with normal inhibition at three to four weeks, suggesting a disrupted termination of the critical period in these mice.

A previous report showed that the number of GABAergic interneurons, including parvalbumin-immunopositive interneurons, is reduced in Cntnap2 KO mice examined at postnatal day 14, indicating a disruption of inhibitory output during early development (Peñagarikano et al., 2011). Our results revealed that Cntnap2 KO mice have normal phasic and tonic inhibition at three to four weeks old. This discrepancy may be explained by recent discoveries which concluded that the reduction of PV-immunopositive interneurons in autism mouse models reflects the downregulation of PV expression, rather than cell loss (Filice et al., 2016; Lauber et al., 2016). Our findings of disrupted inhibition are largely consistent with a previous study in Cntnap2 KO mice at a similar developmental time point, which found alteration of inhibition in hippocampal CA1 pyramidal cells (Jurgensen and Castillo, 2015). While Jurgensen and Castillo did report increased, rather than decreased, sIPSC frequency, this may be attributable to a difference in brain regions and circuit specificity.

The exact mechanism of how the deletion of Cntnap2 causes the reduction of tonic conductance and reduced sIPSC frequency in later development is unclear. It has been shown that Cntnap2 is expressed in presynaptic interneurons (Pinatel et al., 2015), and its expression is greatly increased through postnatal development (Gordon et al., 2016), thus the deletion of Cntnap2 may alter the stability of inhibitory synapses in the later phase of the critical period. Another possibility is reduction of PV expression, which has been reported in several autism mouse models (Gogolla et al., 2009; Filice et al., 2016; Lauber et al., 2016). Because of the tight correlation between the expression of PV and the maturation of inhibitory synapses (del Río et al., 1994; Donato et al., 2013), it appears possible that reduced expression of PV results in the disruption of inhibition after the deletion of Cntnap2. Further investigation is required to test these hypotheses.

Downregulation of tonic GABAergic inhibition was previously reported in subiculum (Curia et al., 2009) and amygdala (Olmos-Serrano et al., 2010; Martin et al., 2014) in Fmr1 KO mice, a mouse model of fragile X syndrome, which also exhibits autism-related behaviors. Fmr1 KO mice show increased phosphorylation of the β3 subunit of GABA_A_R at the S408/9 site that facilitates GABA_A_R endocytosis (Vien et al., 2015). Accordingly, in mice with S408/9A mutation, tonic inhibition is reduced while the amplitude of sIPSCs is increased. Our results demonstrated that tonic inhibition and the frequency, but not the amplitude or kinetics, of sIPSCs, are reduced in Cntnap2 KO mice, which may result from the reduction in the number of inhibitory synapses but not the change in the phosphorylation of β3 subunit.

Altered gamma oscillation and epilepsy have been reported in individuals with autism (Rojas and Wilson, 2014) and in animals with deletion of autism-risk genes, including Cntnap2 KO mice and rats (Peñagarikano et al., 2011; Thomas et al., 2017). Since tonic inhibition is found to be involved in the generation of gamma oscillations (Mann and Mody, 2010) and altered tonic inhibition also contributes to epilepsy (Roberts et al., 2005; Ferando and Mody, 2012; Lee and Liou, 2013), the decreased tonic inhibition in Cntnap2 KO mice observed in the current study suggests an underlying mechanism for those phenotypes in individuals with autism. Consistent with the report that hyperactivity in Fmr1 KO mouse can be rescued by THIP application (Martin et al., 2016), the current study showed that THIP changed the excitability of pyramidal cells, suggesting that THIP may be useful as a potential therapeutic agent in autism.

## Synthesis

Reviewing Editor: Pablo Castillo, Albert Einstein College of Medicine Decisions are customarily a result of the Reviewing Editor and the peer reviewers coming together and discussing their recommendations until a consensus is reached. When revisions are invited, a fact-based synthesis statement explaining their decision and outlining what is needed to prepare a revision will be listed below. The following reviewer(s) agreed to reveal their identity: Alfredo Kirkwood

Your manuscript has been evaluated by two experts in the field. Although their opinions were initially split, the following summarizes their common views on your study. They both appreciate the quality of the results and potential of your study, but they also raise significant concerns and agree that further characterization of the Cntnap2 mouse is required. Specifically, they highlight the following two points:

1) Improve the intrinsic excitability analysis and perform the experiments of Figure 5 also in KO and WT slices obtained from the older age group.

2) Show that the reduction of excitability induced by THIP application (Figure 6) is weaker in KO vs control mice of the older age group (and ideally also show that the difference between genotypes is absent in the younger age group).

In addition to these two main points, you should address all other points raised by the reviewers (see below).

REVIEWER #1:

The study reports 3 new findings concerning the Cntnap2 KO mice, an important model of autism. 1) the deficits in fast GABAergic transmission, previously characterized in the hippocampus, can also be observed in the visual cortex. 2) the deficits are age-dependent and 3) slow, tonic GABA responses are also affected by the genetic ablation.

The experiments are well executed, the results are convincing and the narrative is easy to follow. I think the study is a good fit for the mission of eNeuro.

REVIEWER #2:

This manuscript compares GABAergic signaling onto layer 2/3 pyramidal cells of the visual cortex of control vs. Cntnap2 KO mice. The authors report genotype-related differences both in phasic and tonic GABAergic signaling, but only at relatively mature stages. As the Cntnap2 KO mouse is considered a model of autism spectrum disorders, results are potentially interesting. However, while recordings and traces are very nice, the overall level of analysis remains at a rather superficial level, and does not give the impression of a complete work. In addition, there are a few important points of concern that should be addressed by the authors.

Major points

1) Comparison of intrinsic excitability (Figure 5). First, why was this experiment performed only in 3-4 week old animals and not also in the 6-8 week old group? After all, differences in GABAergic currents were only observed in the latter group. Second, was any difference in membrane resting potential observed between the two genotypes? Third, at what membrane potential(s) were neurons held before delivering current ramps and steps? Fourth, how was action potential threshold exactly assessed? In the text, the "action potential threshold" is measured in pA. I suspect that the authors are actually measuring some sort of rheobase-like index. More methodological details are needed. Finally, the sentence "step-current induced firing rate for all cells was normalized to the AP threshold" is also confusing, as the X axis of Figure 5 F seems to give absolute current values (pA) and the Y axis absolute frequencies (Hz). I am not even sure why this "normalization" would be necessary.

2) If the genotype-related differences in tonic GABAergic currents revealed by the authors in Figure 2 had a significant effect, then one would expect to see differences in neuronal excitability in slices obtained from 6-8 week old control vs Cntnap2 KO mice, at least in the presence of 5 uM GABA as in Figure 2. I think this experiment should be performed.

3) Figure 6. First, the authors do not indicate (either in the text or legend) the age of the animals they used. Most importantly, they should compare the magnitude of the effect of THIP between the two different genotypes and show that there is a reduced effect on Cntnap2 KO mice. Lastly, it is still unclear to me whether Figure 6C indicates absolute values of current injected during the steps (as it would appear from the figure) or some sort of stimulus normalization (as it would appear from the text: "we aligned firing rate versus current injection for all cells to the first current step before AP threshold") and why this would be necessary.

Minor points

1) "the tonic inhibition induced by ambient GABA is not detectable in acute brain slices". Although the authors are correct that detection is harder without pharmacological manipulations (addition of GABA or NO711), tonic GABA currents have been recorded also in drug-free ACSF. Please reword the sentence.

2) Are the peaks of the traces in Figures 2, 3 and 4 capacitive transients in response to voltage steps used to monitor series resistance? Please clarify.

3) The authors state that "the reduced inhibition in 6-8 week old Cntnap2 KO mice in our study is consistent with another previous report [...] (Jurgensen and Castillo, 2015).". I am not sure I would agree with the authors as Jurgensen and Castillo found increased sIPSC freqeuncy in KO mice whereas the authors report the opposite change (decreased frequency).

4) The authors claim that they analyzed the kinetics of sIPSCs, but how this analysis was performed is neither described in the methods section nor are risetime/decay time tau values included in the text.
